# Reading the Evolution of Compartmentalization in the Ribosome Assembly Toolbox: The YRG Protein Family

**DOI:** 10.1371/journal.pone.0169750

**Published:** 2017-01-10

**Authors:** Pablo Mier, Antonio J. Pérez-Pulido, Emmanuel G. Reynaud, Miguel A. Andrade-Navarro

**Affiliations:** 1 Institute of Molecular Biology (IMB), Faculty of Biology, Johannes-Gutenberg University of Mainz, Mainz, Germany; 2 Centro Andaluz de Biologia del Desarrollo (CABD, UPO-CSIC-JA). Facultad de Ciencias Experimentales (Área de Genética), Universidad Pablo de Olavide, Sevilla, Spain; 3 School of Biomolecular and Biomedical Science, University College Dublin, Dublin, Ireland; Laboratoire de Biologie du Développement de Villefranche-sur-Mer, FRANCE

## Abstract

Reconstructing the transition from a single compartment bacterium to a highly compartmentalized eukaryotic cell is one of the most studied problems of evolutionary cell biology. However, timing and details of the establishment of compartmentalization are unclear and difficult to assess. Here, we propose the use of molecular markers specific to cellular compartments to set up a framework to advance the understanding of this complex intracellular process. Specifically, we use a protein family related to ribosome biogenesis, YRG (YlqF related GTPases), whose evolution is linked to the establishment of cellular compartments, leveraging the current genomic data. We analyzed orthologous proteins of the YRG family in a set of 171 proteomes for a total of 370 proteins. We identified ten YRG protein subfamilies that can be associated to six subcellular compartments (nuclear bodies, nucleolus, nucleus, cytosol, mitochondria, and chloroplast), and which were found in archaeal, bacterial and eukaryotic proteomes. Our analysis reveals organism streamlining related events in specific taxonomic groups such as Fungi. We conclude that the YRG family could be used as a compartmentalization marker, which could help to trace the evolutionary path relating cellular compartments with ribosome biogenesis.

## Introduction

The origin of cellular compartmentalization has been subject of study using molecular evolution now for more than thirty years [1]. Mitochondria and chloroplast have been clearly rooted within the alpha-proteobacteria and cyanobacteria, respectively [2–4]; on the other hand, to explain the origin of eukaryotes different theories have been proposed [5–9], while the explanation of a simple fusion or endosymbiosis involving two prokaryotes has been favored to explain the dual nature of the eukaryotic genome and compartmentalized structure of the eukaryotic cell [8].

Genomic analyses have been extensively used to support different theories of eukaryotic compartmentalization evolution based on a specific set or subset of genes often related to rRNA sequences, but without any link to compartments or compartmentalization events. The cellular machinery related to rRNA molecules is a possible source for molecular markers that could be associated to compartments since this machinery must be present in nearly every cellular compartment from nuclear bodies, nucleolus, nucleus and cytosol, to the endomembrane system, ensuring the essential coupling between translation and transcription.

Following this idea, we characterized the YRG protein family (YlqF Related GTPases). This is a GTP protein binding family composed of key proteins involved in 60S ribosome subunit biogenesis and maintenance [10–13]. Members of this family contain a unique central circularly permutated MMR/HSRI GTPase domain [14,15]. The YRG family was reported to have nine subfamilies represented by nine proteins: YlqF, YjeQ, Noa1, Mtg1, Lsg1, Gnl1, Gnl2, Gnl3l and Gnl3 [14], with different subcellular locations. We propose here a tenth YRG protein subfamily, named YAG (YRG Archaeal GTPases).

Because YRG proteins are necessary for the rRNA assembly activity in different cellular compartments, it is generally expected that each YRG protein will be only present in a given organism within a specific subcellular compartment; thus, following up the evolution of YRG proteins across subcellular compartments and taxa would allow following the corresponding evolution of compartments.

To illustrate the use of the YRG family as such markers of compartment evolution, we analyzed orthologous proteins of the family in a set of 171 proteomes (32 Bacteria, 93 Archaea and 46 Eukarya) and found a total of 370 proteins. Our analysis reproduced the major events of the evolution of eukaryotic compartmentalization, supporting the YRG protein family as a reliable compartmentalization tracer, able to predict compartment schemes in an evolutionary wide range of organisms.

## Methods

### Data retrieval

A total of 171 reference proteomes with a complete set of sequences and functional annotations were downloaded from the database UniProt release 2015_05 [16] (S1 File). The canonical sequence dataset from each proteome was used. The proteomes covered a wide taxonomic range: 32 bacterial, 93 archaeal and 46 eukaryotic.

### Search for YRG proteins

The YRG search was performed using the standalone version of orthoFind with default parameters [17] and well-annotated YRG proteins as query sequences. It starts with an exhaustive and iterative local PSI-BLAST search, combined with a reciprocal best-hit protein BLAST (RBHB) strategy, which allows the finding of orthologous proteins from an initial seed sequence. Each result was manually checked to avoid assigning proteins to two different ortholog groups. Ortholog absences were initially checked by a manual RBHB search (seed versus database, reviewing all the significant hits), and secondly with a search in two different orthology repositories: OrthoDB [18] and EggNOG [19].

The following well-annotated YRG proteins were used as seed sequences to search for orthologous proteins in the 171 selected proteomes: *Bacillus subtilis* (YlqF: O31743; Noa1: P54453), *Escherichia coli* (YjeQ: P39286), *Sulfolobus solfataricus* (SSO0581: Q7LXT6) and *Homo sapiens* (Mtg1: Q9BT17; Lsg1: Q9H089; Gnl1: P36915; Gnl2: Q13823; Gnl3l: Q9NVN8; Gnl3: Q9BVP2). Query sequence data was obtained from the UniProt Knowledgebase [16]. Accession number (AC) and subcellular location of the YRG sequences were obtained from their UniProt file.

### Phylogenetic analysis

The 370 YRG proteins we found were used to construct a multiple sequence alignment (S1 File), since they correspond to the functional sequences. To do that, we used MAFFT v7.205–1 [20], which presents a high accuracy aligning datasets with low global similarity. Since the YRG protein family has a complex motif architecture, the *linsi* options were used (L-INS-i and iterative refinement method:—localpair—maxiterate 1000), based on its accuracy with multi-motif proteins [21].

The multiple sequence alignment was used to build a molecular phylogeny with PhyML [22], which presents reliable results for large data sets with high sequence divergence. Since the multiple alignment had long gap regions (intervals longer than 20 positions), positions with residues from less than 10 sequences were cut off. Then, we used ProtTest to find WAG as the best amino acid replacement model, with a confidence interval of 100. Therefore, we built the phylogeny with PhyML, the WAG model and 1000 bootstrap replicates to measure the support of the tree branches. The phylogeny was edited using the java version of the FigTree software (http://tree.bio.ed.ac.uk/software/figtree/).

## Results and Discussion

### The presence of YRG proteins is linked to specific subcellular locations

**Fig 1:**
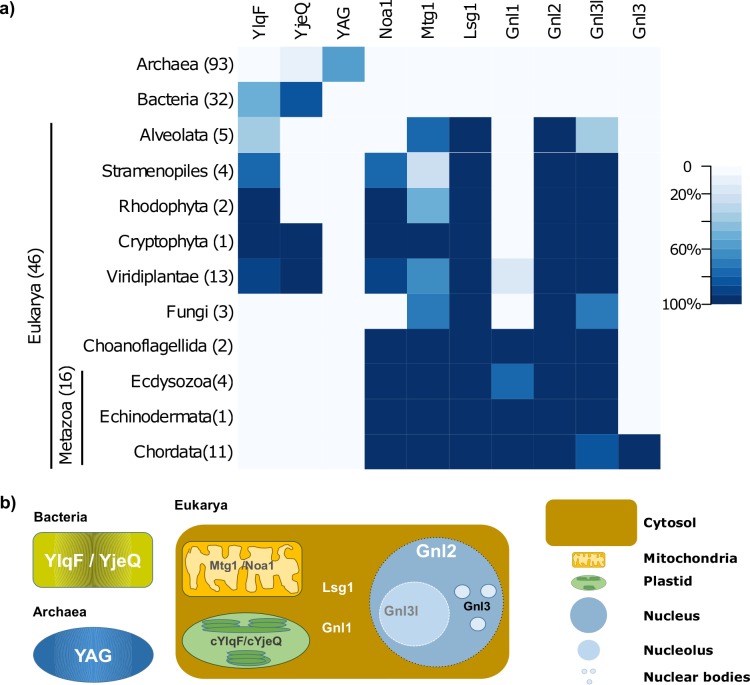
Presence of YRG proteins in 171 proteomes from different taxonomic groups, and their subcellular location. a) Heat map showing the number of species where members of the different YRG subfamilies were detected. b) Subcellular locations assigned to each of the ten YRG protein subfamilies, in Bacteria, Archaea and Eukarya, based on annotations from the well-annotated sequences used as seed for the search.

Taking advantage of the growing set of proteins in the databases, we studied the distribution of YRG family members across the tree of life, in a wide set of taxonomic groups and subcellular compartments. We searched 171 organisms (32 Bacteria, 93 Archaea and 46 Eukarya) for YRG sequences, using both tools to find orthologs (orthoFind [17] and RBHB) and databases of orthologous proteins (OrthoDB [18] and EggNOG [19]) (see Methods). The number of putative YRG proteins found was 370, spread over the different taxonomic divisions (Fig 1A; S1 File; S4 File).

Bacteria have a maximum of two YRG proteins (YlqF and YjeQ). Both are broadly conserved in bacteria and have been shown to be essential for their growth [23,24]. Notably, from them only YjeQ is found in eight out of the 93 studied archaeal proteomes, all of which belong to the phylum Euryarchaeota, class Methanomicrobia.

Each archaeal proteome has a maximum of one YRG protein (with the exception of those that also harbour bacterial YjeQ), which we name YAG (YRG Archaeal GTPase). As archaea have no subcellular compartments, its ribosomal activity is restricted to the cytosol [25]. Thus, the presence of just one YRG protein in most archaeal organisms is coherent with the number of cellular locations with ribosomal activity.

Eukaryotes have up to seven YRG proteins (Fig 1A; S1 File). All of the known YRG proteins are present in at least one eukaryotic taxa except for YAG, a typically-archaeal protein. Those proteins are restricted to specific subcellular compartments, which also correlate with the taxonomy of the studied proteomes (Fig 1B). For example, plants and other species with plastids like the algae *Gillardia theta* have the bacterial proteins, cYlqF and cYjeQ, as a result of the acquisition of the plastid via an endosymbiotic event [14]. Similarly, the proteins Mtg1 and Noa1, present in the majority of eukaryotic proteomes, are similar to the bacterial YlqF and YjeQ, respectively, and are located in mitochondria [26–28], in agreement to the endosymbiotic origin of mitochondria [29].

Lsg1 is present in all of the 46 studied eukaryotic proteomes, and is located mainly in the cytosol but shuttling to the nucleus upon specific events (Fig 1B) [14,30,31]. Its subcellular location is similar to Gnl1, another YRG protein which is localized mainly in the cytosol while shuttling to nucleus and nucleolus in the cell cycle stage G2 [32]. Finally, three proteins are restricted to the nuclear compartment and intra subcompartments: Gnl2, present in all of the eukaryotic proteomes and shuttling between nucleus and nucleolus [33]; Gnl3l, absent in some Alveolata proteomes and specific to the nucleolus [34], and Gnl3, also known as Nucleostemin, only present in Chordata [35].

### Correlation between the evolution of the YRG proteins and their subcellular location

As already described, YRG proteins are characterized by their linkage to specific subcellular compartments. Furthermore, they are related to each other, as they all evolved from a same YRG ancestral protein. To clarify their evolutionary history and see how this correlates with the evolution of compartmentalization, we conducted a phylogenetic analysis using the complete dataset of YRG proteins.

**Fig 2:**
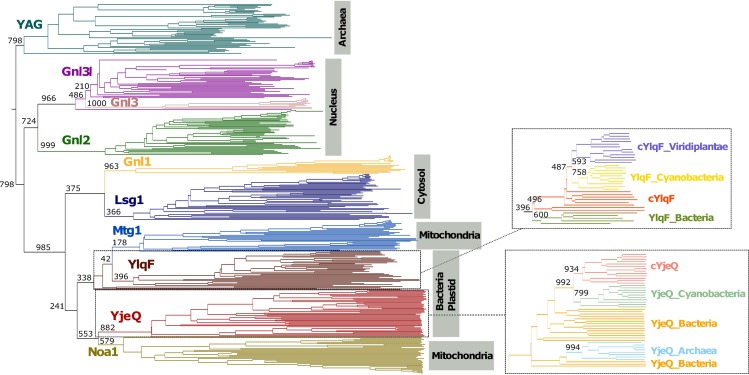
Phylogeny of the 370 YRG proteins found in the analyses. The sequences are disposed in ten branches, one for each YRG protein subfamily: Gnl3, Gnl3l, Gnl2, Lsg1, Gnl1, YlqF, Mtg1, YjeQ, Noa1 and YAG. Main branches are labeled with a bootstrap support value (0–1000) (see Methods for details). Branches are annotated with information about the subcellular location of the subfamily. Zoom-in of YlqF and YjeQ clades are shown to describe their diversity in different taxonomic groups.

The phylogenetic tree shows that all archaeal YRG proteins cluster together in the same branch of the tree (Fig 2; raw tree file in S2 File). This supports YAG as a separate subfamily of the YRG family. All YjeQ-like proteins present in archaea appear in a separate branch clustered together with the rest of the YjeQ proteins, in agreement with an event of horizontal transfer of this subfamily to archaea (Fig 2).

Interestingly, the YRG subfamilies seem to be polarized in either bacterial-origin or eukaryotic-specific subfamilies, as shown in the phylogeny (Fig 2). This suggests that all eukaryotic members originated from a common ancestor. Within these eukaryotic families two clear branches appear grouping the cytosolic (Lsg1 and Gnl1) and the nuclear (Gnl3, Gnl3l, Gnl2) subfamilies. The positioning of the Gnl3 subfamily within the Gnl3l branch, as well as its restricted presence to Chordata organisms, suggests a late evolutionary appearance, in a subcellular location closely related to that of its parental gene.

Families of bacterial origin are in a branch both with bacterial YlqF (mitochondrial Mtg1) and YjeQ (mitochondrial Noa1), each including one plastid member (cYlqF and cYjeQ). The plastid protein clades are grouped with cyanobacterial proteins (Fig 2), as expected due to the cyanobacterial origin of plastids [36].

### The YRG evolution scheme supports the evolution of compartmentalization in eukaryotes

**Fig 3:**
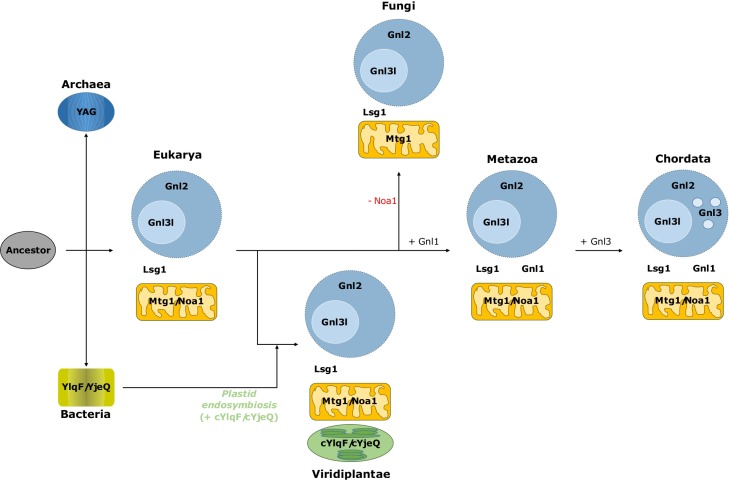
Overview of the evolutionary history of the YRG family along with the history of the compartmentalization in eukaryotes. This YRG evolution model was established based on the presence of the different YRG proteins in 171 proteomes from different taxonomic groups (S1 File), their subcellular locations (Fig 1B) and the relations between the YRG proteins inferred from the phylogenetic tree (Fig 2).

Cells require at least one YRG protein per compartment in regard to rRNA assembly activity. By further correlating the presence of YRG proteins in 171 proteomes from different taxonomic groups (Fig 1A), the subcellular localization information for each YRG protein (Fig 1B), and the relations between them inferred from the phylogenetic data (Fig 2), we constructed a detailed picture of the YRG evolution scheme that corresponds to the evolution of compartmentalization in eukaryotes (Fig 3).

The ancestral “single compartment” YRG protein would have led to both the archaeal YAG and to an ancestral protein of bacterial YlqF and YjeQ. Regarding eukaryotes, results show that all of them contain both Lsg1 and Gnl2. This suggests that within the first eukaryotes, Gnl2 was involved in the biogenesis of the 60S ribosome subunit within the nucleus [33] while Lsg1 performed probably a similar function further down the rRNA biogenesis pathway within the cytosol [12].

Gnl1 is a cytosolic YRG protein restricted mostly to metazoans (Fig 1A). The fact that it shuttles both to nucleus and nucleolus [32] suggests its evolution related to the increase in complexity of the nuclear compartment. While the most parsimonious explanation for the evolution of Gnl1 is that it emerged as a duplication of Lsg1 in the metazoan lineage (Fig 3), our phylogeny does not support this as the Gnl1 subfamily clusters outside Lsg1 suggesting that it was lost in Fungi and Viridiplantae.

Besides Gnl2, the other proteins in the nuclear compartment are Gnl3l and Gnl3. Gnl3l is almost as prevalent in eukaryotes as Gnl2 (Fig 1A); its nucleolar location and wide taxonomic distribution hints that it duplicated from Gnl2 as part of the emergence of the nucleolus. Gnl3, localized in the nuclear bodies and present only in chordates, is the most recent YRG protein and appeared as a duplication of Gnl3l (Fig 2). The nucleolus has kept evolving along the evolution of chordates. For example, in Amniota (reptiles and mammals) the nucleolus has three subcompartments, instead of the two present in the rest of the eukaryotes [37,38]. The emergence of Gnl3 might have facilitated this evolution (Fig 2), complementing the function of Gnl2 and Gnl3l in the nuclear/nucleolar ribosomal biogenesis and maintenance.

While the sequence similarity of Mtg1 and Noa1 with the two bacterial YRG proteins (i.e. YlqF and YjeQ) would seem to agree with their acquisition due to the mitochondrial endosymbiosis event, their position in our phylogenetic tree (Fig 2) does not support it for YjeQ/Noa1 as they both are coming from monophyletic branches. But YlqF constitutes a paraphyletic group, with Mtg1 diverging from it, which supports the endosymbiotic event. Furthermore, the gaining by Viridiplantae species of two more YRG proteins (cYlqF and cYjeQ), responding to the endosymbiotic event that led to the acquisition of chloroplast by a non-photosynthetic eukaryotic organism [36], is also supported by our phylogenetic tree, at least in the case of YjeQ. Cyanobacteria, as most bacteria, have only two YRG proteins, the aforementioned YlqF and YjeQ; the YRG phylogenetic tree (Fig 2) suggests the branching of cyanobacterial YlqF and YjeQ with their plastid counterparts (high bootstrapping values in Fig 2). Nevertheless, we must highlight the low support of all these branches in our phylogeny, in contrast to the branch of archaebacteria and nucleus, which have been independently confirmed by another phylogenetic method (S5 File), which does not show the paraphyletic relation for YlqF. The conclusions should be interpreted with caution, given the low support of the tree, due to the high divergence of the YRG proteins in the different compartments.

When focusing on opisthokonts (represented in our analysis mainly by fungal and metazoan proteomes), it seems that almost all of them contain two nuclear-nucleolar proteins (Gnl2 and Gnl3l), one cytosolic (Lsg1), and two mitochondrial (Mtg1 and Noa1). However, none of the fungal proteomes presents Noa1, while other opisthokonts such as choanoflagellids and metazoans do have Noa1 orthologs, as well as taxa that appeared prior to the emergence of opisthokonts (plants, for example). Fungi would have lost the *noa1* gene in a gene loss event that appears to be specific to this taxonomical class.

As stated before, we expect to find members of the YRG family in cellular compartments with ribosomal activity, namely nuclear bodies, nucleolus, nucleus, cytosol, mitochondria and plastids. Accordingly, we do not observe extra YRG proteins in organisms such as bacteria *Magnetospirillum magneticum* or *Magnetobacterium bavaricum*. These organisms have magnetosomes [39], subcellular structures for magnetotaxis, which have associated proteins but no described ribosomal activity. Conversely, we predict the loss of particular YRG proteins in organisms lacking the subcellular compartment to which they relate. One way to observe this phenomenon is looking at parasites, e.g. the microsporidian *Encephalitozoon cuniculi*. Although it is a fungal organism, *E*. *cuniculi* is an obligate intracellular parasite with a minimal genome among eukaryotes [40]. Unlike the rest of the fungi, *E*. *cuniculi* has neither Mtg1 nor Gnl3l. As an intracellular parasite, it uses the host's cellular machinery and therefore does not have mitochondria [40], turning Mtg1 into a non-essential protein. The absence of Gnl3l in this organism responds to not having a complex nucleolus. Similarly, the parasite *Cryptosporidium parvum* (Alveolata) has neither Mtg1 nor Noa1, although it does contain a mitochondrion-like organelle without mitochondrial genome [41]. As such unusual mitochondria import all necessary proteins from the cytoplasm [42], they would not require rRNA assembly proteins explaining the absence of YRG proteins.

The evolution of the YRG protein family provides a cell biological evolutionary line for the compartmentalization of eukaryotic cells. The presence of YRG proteins serves as a compartmentalization marker that can be used to infer evolution events for the whole of eukarya and for specific taxa evolution (e.g. Fungi).

## Conclusions

To understand the origin and evolution of compartmentalization in eukaryotic cells, we used the YRG (YlqF related GTPases) protein family as a molecular marker. This family was reported to be composed of nine subfamilies and found specifically in six subcellular compartments. The study of YRG proteins in a wide set of proteomes led us to propose the existence of an archaeal specific subfamily, which we named YAG (YRG Archaeal GTPase). We propose therefore that the YRG protein family is composed of ten subfamilies functioning in different subcellular locations: YlqF (bacteria and plastids, cYlqF), YjeQ (bacteria and plastids, cYjeQ), YAG (archaea), Noa1 (mitochondria), Mtg1 (mitochondria), Lsg1 (cytosol), Gnl1 (cytosol), Gnl2 (nucleus), Gnl3l (nucleolus) and Gnl3 (nuclear bodies). Association of YRG protein subfamilies to specific subcellular compartments and taxa allowed us to use the YRG family as an indicator of the evolution of cellular compartments. Moreover, as the YRG family is related to ribosome biogenesis and maintenance, it represents a functional ribosome biogenesis marker rather than an rRNA sequence tracer.

## Supporting Information

S1 File Set of homologous proteins of the YRG protein family. A total of 171 proteomes were used: 32 Bacteria, 93 Archaea and 46 Eukarya. The dash symbol “-” means the absence of the protein in that proteome. If the protein is present in a proteome, the UniProt Accession Number (AC) is shown.
(XLSX)

S2 File Raw file of the phylogenetic tree obtained with PhyML, with bootstrapping values (0–1000), in Newick format. Each sequence is labeled using the YRG protein, the species name and the taxonomical group it belongs to: YRGprotein_Organism_Phyla.
(TXT)

S3 File Phylogenetic trees (one per YRG subfamily) obtained with PhyML. Each sequence is labeled using the YRG protein, the species name and the taxonomical group it belongs to: YRGprotein_Organism_Phyla.
(PDF)

S4 File Set of homologous proteins of the YRG protein family, in FASTA format. Each sequence is labeled using the YRG protein and the species name: YRGprotein_Organism.
(FASTA)

S5 File Phylogeny of the 370 YRG proteins found in the analyses using an alternative method (RAxML). The sequences are disposed in ten branches, one for each YRG protein subfamily: Gnl3, Gnl3l, Gnl2, Lsg1, Gnl1, YlqF, Mtg1, YjeQ, Noa1 and YAG. Main branches are labeled with a bootstrap support value (0–100), and the lowly supported ones are highlighted in red color.
(PDF)

## References

[1] Gabaldon T, Pittis AA. Origin and evolution of metabolic sub-cellular compartmentalization in eukaryotes. Biochimie. 2015;119: 262–268. 10.1016/j.biochi.2015.03.021 25869000 PMC4678951

[2] Esser C, Ahmadinejad N, Wiegand C, Rotte C, Sebastiani F, Gelius-Dietrich G, et al A genome phylogeny for mitochondria among alpha-proteobacteria and a predominantly eubacterial ancestry of yeast nuclear genes. Mol Biol Evol. 2004;21: 1643–1660. 10.1093/molbev/msh160 15155797

[3] Archibald JM. The puzzle of plastid evolution. Curr Biol. 2009;19: 81–88. 10.1016/j.cub.2008.11.067 19174147

[4] Elias M, Archibald JM. Sizing up the genomic footprint of endosymbiosis. Bioessays. 2009;31: 1273–1279. 10.1002/bies.200900117 19921698

[5] Poole AM, Penny D. Evaluating hypotheses for the origin of eukaryotes. Bioessays. 2007;29: 74–84. 10.1002/bies.20516 17187354

[6] Gribaldo S, Poole AM, Daubin V, Forterre P, Brochier-Armanet C. The origin of eukaryotes and their relationship with the Archaea: are we at a phylogenomic impasse? Nature Reviews Microbiology. 2010;8: 743–752. 10.1038/nrmicro2426 20844558

[7] Cavalier-Smith T. Origin of the cell nucleus, mitosis and sex: roles of intracellular coevolution. Biol Direct. 2010;5. 10.1186/1745-6150-5-7 PMC2837639 20132544

[8] Koonin E.V. The origin and early evolution of eukaryotes in the light of phylogenomics. Genome Biol. 2010;11. 10.1186/gb-2010-11-5-209 PMC2898073 20441612

[9] Forterre P. A new fusion hypothesis for the origin of Eukarya: better than previous ones, but probably also wrong. Research in Microbiology. 2011;162: 77–91. 10.1016/j.resmic.2010.10.005 21034817

[10] Kim J, Jang JY, Yoon HJ, Suh SW. Crystal structure of YlqF, a circularly permuted GTPase: implications for its GTPase activation in 50 S ribosomal subunit assembly. Proteins. 2008;72: 1363–1370. 10.1002/prot.22112 18536017

[11] Anand B, Surana P, Bhogaraju S, Pahari S, Prakash B. Circularly permuted GTPase YqeH binds 30S ribosomal subunit: Implications for its role in ribosome assembly. Biochem Biophys Res Commun. 2009;386: 602–606. 10.1016/j.bbrc.2009.06.078 19540197 PMC2741578

[12] Kallstrom G, Hedges J, Johnson A. The putative GTPases Nog1p and Lsg1p are required for 60S ribosomal subunit biogenesis and are localized to the nucleus and cytoplasm, respectively. Mol Cell Biol. 2003;23: 4344–4355. 10.1128/MCB.23.12.4344-4355.2003 12773575 PMC156149

[13] Saveanu C, Namane A, Gleizes PE, Lebreton A. Rousselle JC, Noaillac-Depeyre J, et al Sequential protein association with nascent 60S ribosomal particles. Mol Cell Biol. 2003;13: 4449–4460. 10.1128/MCB.23.13.4449-4460.2003 PMC164837 12808088

[14] Reynaud EG, Andrade MA, Bonneau F, Ly TBN, Knop M, Scheffzek K, Pepperkok R. Human Lsg1 defines a family of essential GTPases that correlates with the evolution of compartmentalization. BMC Biol. 2005;3. 10.1186/1741-7007-3-21 PMC1262696 16209721

[15] Leipe DD, Wolf YI, Koonin EV, Aravind L. Classification and Evolution of P-loop GTPases and Related ATPases. J Mol Biol. 2002;317: 41–72. 10.1006/jmbi.2001.5378 11916378

[16] The UniProt Consortium. UniProt: a hub for protein information. Nucleic Acids Res. 2015;43: 204–212. 10.1093/nar/gku989 PMC4384041 25348405

[17] Mier P, Andrade-Navarro MA, Pérez-Pulido AJ. orthoFind facilitates the discovery of homologous and orthologous proteins. PLoS One. 2015;10. 10.1371/journal.pone.0143906 PMC4666658 26624019

[18] Kriventseva EV, Tegenfeldt F, Petty TJ, Waterhouse RM, Simao FA, Pozdnyakov IA, et al OrthoDB v8: update of the hierarchical catalog of orthologs and the underlying free software. Nucleic Acids Res. 2015;43: 250–256. 10.1093/nar/gku1220 PMC4383991 25428351

[19] Huerta-Cepas J, Szklarczyk D, Forslund K, Cook H, Heller D, Walter MC, et al eggNOG 4.5: a hierarchical orthology framework with improved functional annotations for eukaryotic, prokaryotic and viral sequences. Nucleic Acids Res. 2016;44: 286–293. 10.1093/nar/gkv1248 PMC4702882 26582926

[20] Katoh K, Standley DM. MAFFT multiple sequence alignment software version 7: improvements in performance and usability. Mol Biol Evol. 2013;30: 772–780. 10.1093/molbev/mst010 23329690 PMC3603318

[21] Thompson JD, Linard B, Lecompte O, Poch O. A comprehensive benchmark study of multiple sequence alignment methods: current challenges and future perspectives. PLoS One. 2011;6. 10.1371/journal.pone.0018093 PMC3069049 21483869

[22] Guindon S, Dufayard JF, Lefort V, Anisimova M, Hordijk W, Gascuel O. New algorithms and methods to estimate maximum-likelihood phylogenies: assessing the performance of PhyML 3.0. Syst Biol. 2010;59: 307–321. 10.1093/sysbio/syq010 20525638

[23] Daigle DM, Rossi L, Berghuis AM, Aravind L, Koonin EV, Brown ED. YjeQ, an essential, conserved, uncharacterized protein from Escherichia coli, is an unusual GTPase with circularly permuted G-motifs and marked burst kinetics. Biochemistry. 2002;41: 11109–11117. 12220175 10.1021/bi020355q

[24] Uicker WC, Schaefer L, Britton RA. The essential GTPase RbgA (YlqF) is required for 50S ribosome assembly in Bacillus subtilis. Mol Microbiol. 2006;59: 528–540. 10.1111/j.1365-2958.2005.04948.x 16390447

[25] Evguenieva-Hackenberg E, Roppelt V, Lassek C, Klug G. Subcellular localization of RNA degrading proteins and protein complexes in prokaryotes. RNA Biology. 2011;8:49–54. 10.4161/rna.8.1.14066 21289488 PMC3127078

[26] Barrientos A, Korr D, Barwell KJ, Sjulsen C, Gajewski CD, Manfredi G, et al MTG1 codes for a conserved protein required for mitochondrial translation. Mol Biol Cell. 2003;14: 2292–2302. 10.1091/mbc.E02-10-0636 12808030 PMC194879

[27] Tang T, Zheng B, Chen SH, Murphy AN, Kudlicka K, Zhou H, et al hNOA1 interacts with complex I and DAP3 and regulates mitochondrial respiration and apoptosis. J Biol Chem. 2009;284: 5414–5424. 10.1074/jbc.M807797200 19103604 PMC2643507

[28] Kotani T, Akabane S, Takeyasu K, Ueda T, Takeuchi N. Human G-proteins, ObgH1 and Mtg1, associate with the large mitochondrial ribosome subunit and are involved in translation and assembly of respiratory complexes. Nucleic Acids Res. 2013;41: 3713–3722. 10.1093/nar/gkt079 23396448 PMC3616715

[29] Thrash JC, Boyd A, Huggett MJ, Grote J, Carini P, Yoder RJ, et al Phylogenomic evidence for a common ancestor of mitochondria and the SAR11 clade. Sci Rep. 2011;1. 10.1038/srep00013 PMC3216501 22355532

[30] Weis BL, Missbach S, Marzi J, Bohnsack MT, Schleiff E. The 60S associated ribosome biogenesis factor LSG1-2 is required for 40S maturation in Arabidopsis thaliana. Plant J. 2014;80: 1043–1056. 10.1111/tpj.12703 25319368

[31] Zhao H, Lü S, Li R, Chen T, Zhang H, Cui P, et al The Arabidopsis gene DIG6 encodes a large 60S subunit nuclear export GTPase 1 that is involved in ribosome biogenesis and affects multiple auxin-regulated development processes. J Exp Bot. 2015;66: 6863–6875. 10.1093/jxb/erv391 26272902 PMC4623693

[32] Boddapati N, Anbarasu K, Suryaraja R, Tendulkar AV, Mahalingam S. Subcellular distribution of the human putative nucleolar GTPase GNL1 is regulated by a novel arginine/lysine-rich domain and a GTP binding domain in a cell cycle-dependent manner. J Mol Biol. 2012;416: 346–366. 10.1016/j.jmb.2011.12.066 22244851

[33] Scherl A, Coute Y, Deon C, Calle A, Kindbeiter K, Sanchez JC, et al Functional proteomic analysis of human nucleolus. Mol Biol Cell. 2002;13: 4100–4109. 10.1091/mbc.E02-05-0271 12429849 PMC133617

[34] Rao MRKS, Kumari G, Balasundaram D, Sankaranarayanan R, Mahalingam S. A novel lysine-rich domain and GTP binding motifs regulate the nucleolar retention of human guanine nucleotide binding protein, GNL3L. J Mol Biol. 2006;364: 637–654. 10.1016/j.jmb.2006.09.007 17034816

[35] Ma H, Pederson T. Nucleostemin: a multiplex regulator of cell-cycle progression. Trends Cell Biol. 2008;18: 575–579. 10.1016/j.tcb.2008.09.003 18951797

[36] Gould SB, Waller RF, McFadden GI. Plastid evolution. Annu Rev Plant Biol. 2008;59:491–517. 10.1146/annurev.arplant.59.032607.092915 18315522

[37] Thiry M, Lafontaine D. Birth of a nucleolus: the evolution of nucleolar compartments. Trends Cell Biol. 2005;15: 194–199. 10.1016/j.tcb.2005.02.007 15817375

[38] Lamaye F, Galliot S, Alibardi L, Lafontaine DLJ, Thiry M. Nucleolar structure across evolution: The transition between bi- and tricompartmentalized nucleoli lies within the class Reptilia. J Struct Biol. 2011;174: 352–359. 10.1016/j.jsb.2011.02.003 21335089

[39] Bazylinski DA, Frankel RB. Magnetosome formation in prokaryotes. Nature Rev Microbiol. 2004;2: 217–230. 15083157 10.1038/nrmicro842

[40] Katinka MD, Duprat S, Cornillot E, Méténier G, Thomarat F, Prensier G, et al Genome sequence and gene compaction of the eukaryote parasite Encephalitozoon cuniculi. Nature. 2001;414: 450–453. 10.1038/35106579 11719806

[41] Putignani L, Tait A, Smith HV, Horner D, Tovar J, Tetley L, et al Characterization of a mitochondrion-like organelle in Cryptosporidium parvum. Parasitology. 2004;129: 1–18. 15267107 10.1017/s003118200400527x

[42] Henriquez FL, Richards TA, Roberts F, McLeod R, Roberts CW. The unusual mitochondrial compartment of Cryptosporidium parvum. Trends in Parasitology. 2005;21: 68–74. 10.1016/j.pt.2004.11.010 15664529

