# Supplementary material for: Reading the Evolution of Compartmentalization in the Ribosome Assembly Toolbox: The YRG Protein Family
Source: PLoS One. 2017 Jan 10;12(1):e0169750. doi: 10.1371/journal.pone.0169750 (PMC5224878; doi:10.1371/journal.pone.0169750)
Supplement: S3 File — Each sequence is labeled using the YRG protein, the species name and the taxonomical group it belongs to: YRGprotein_Organism_Phyla. (PDF) [file pone.0169750.s003.pdf]

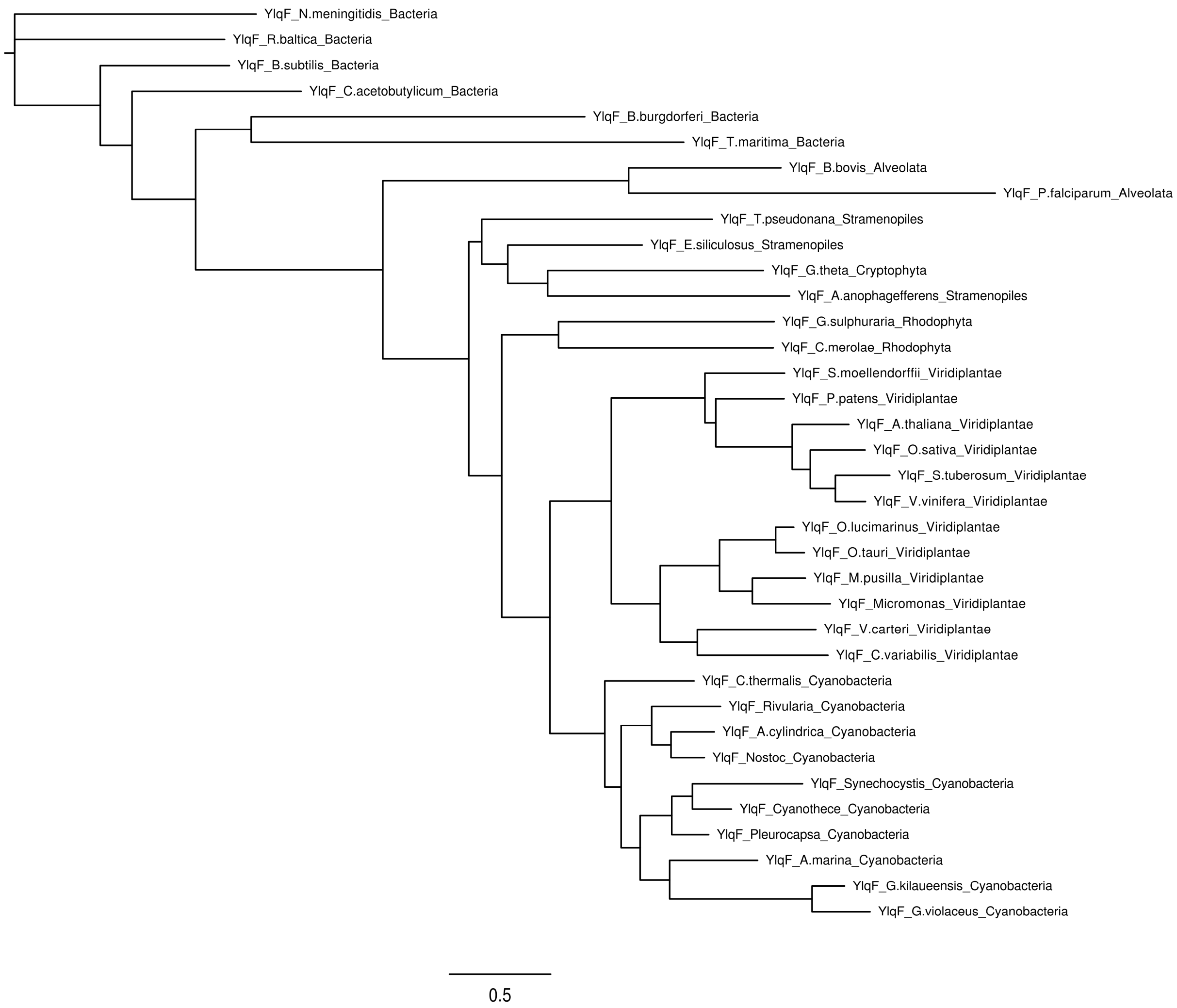

1) YlqF subfamily tree

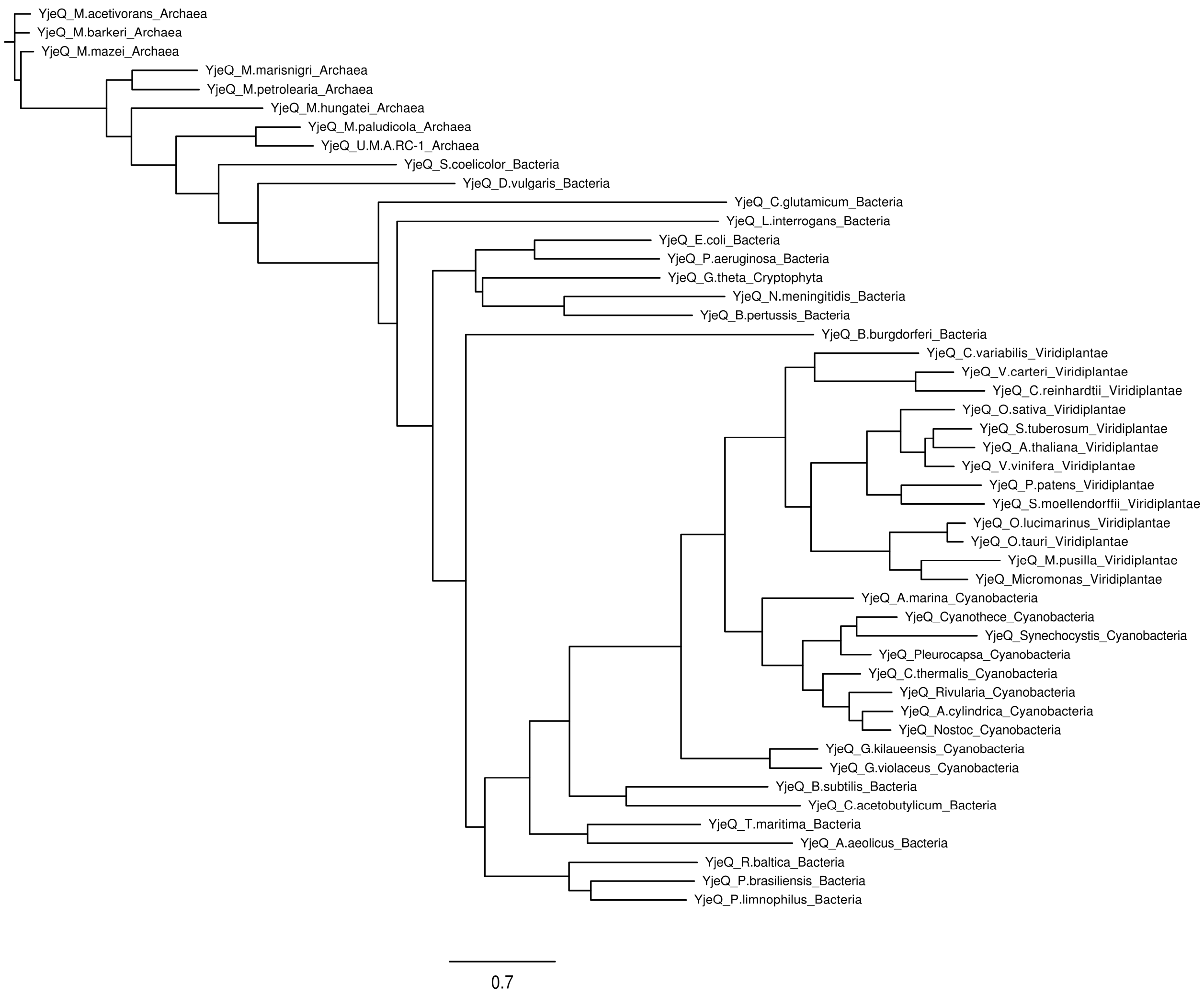

2) **YjeQ** subfamily tree

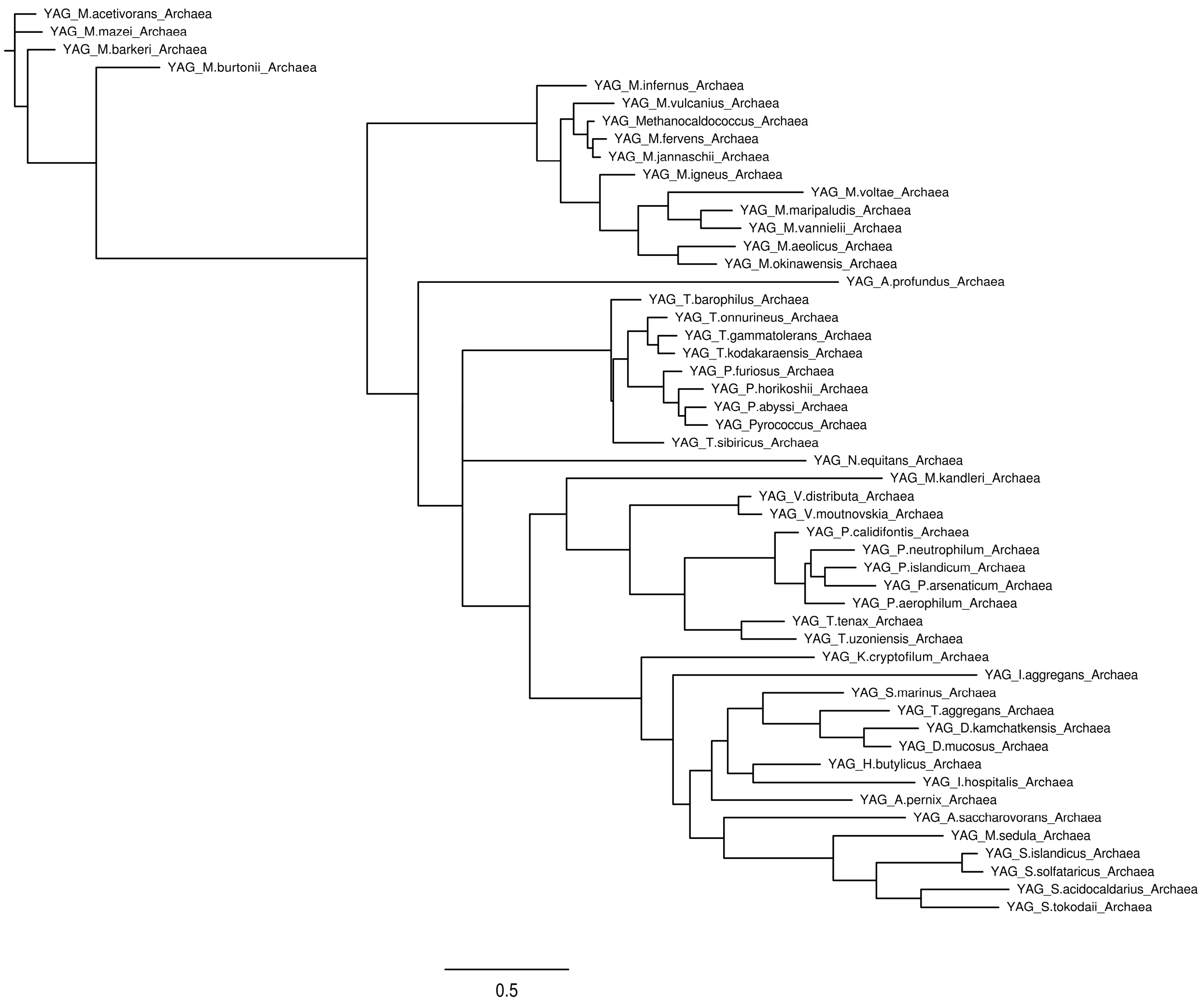

3) **YAG** subfamily tree

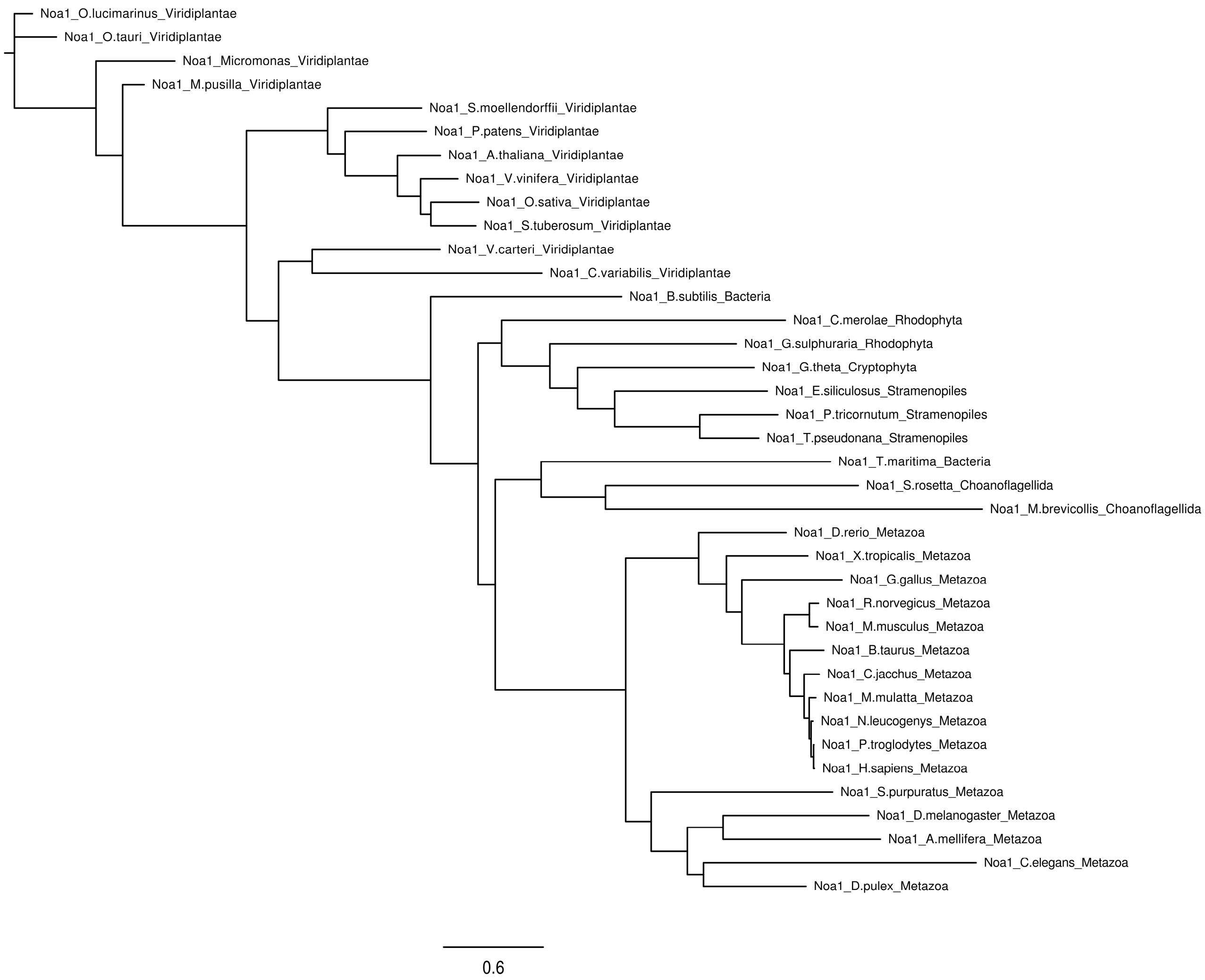

4) **Noa1** subfamily tree

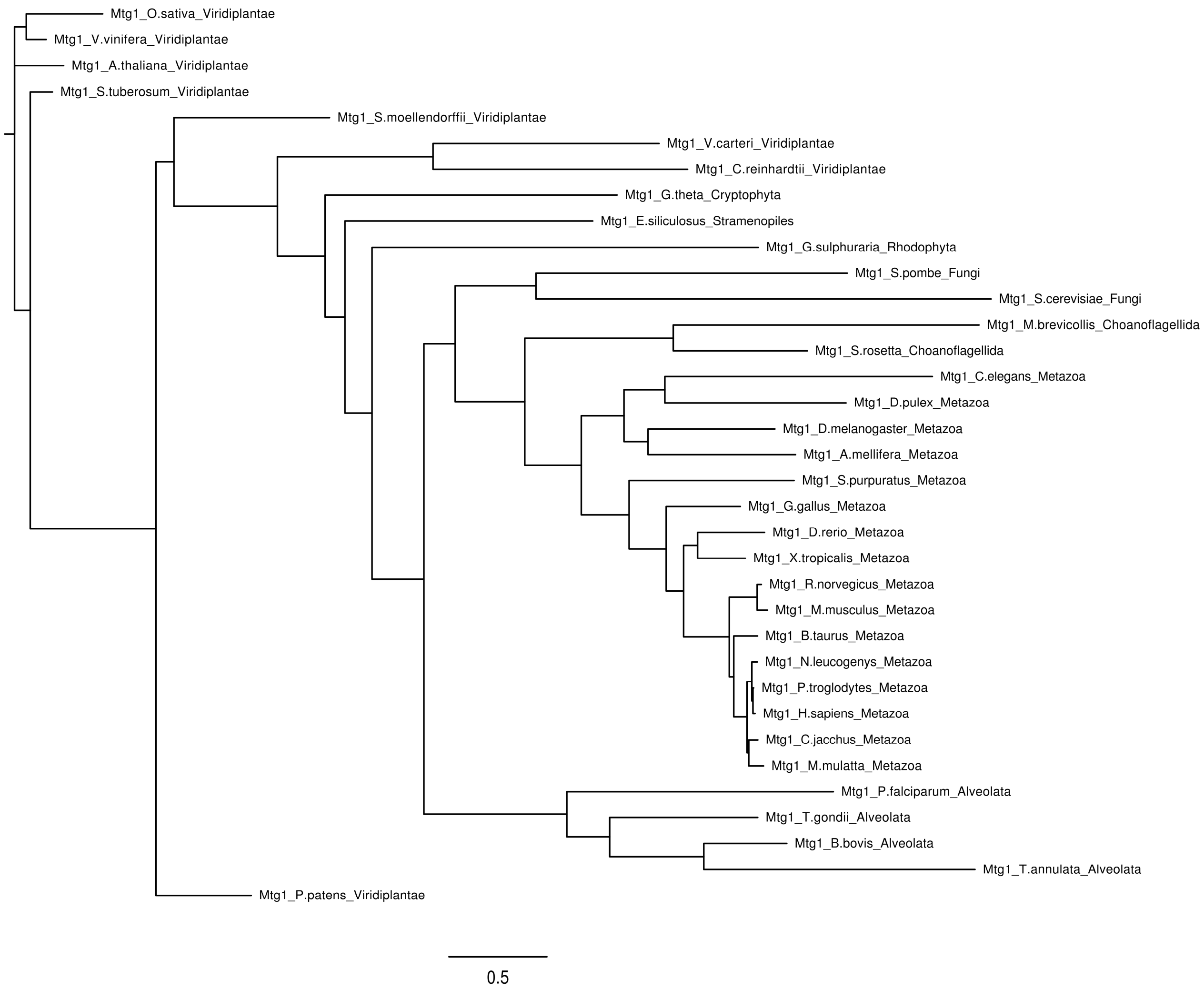

5) **Mtg1** subfamily tree

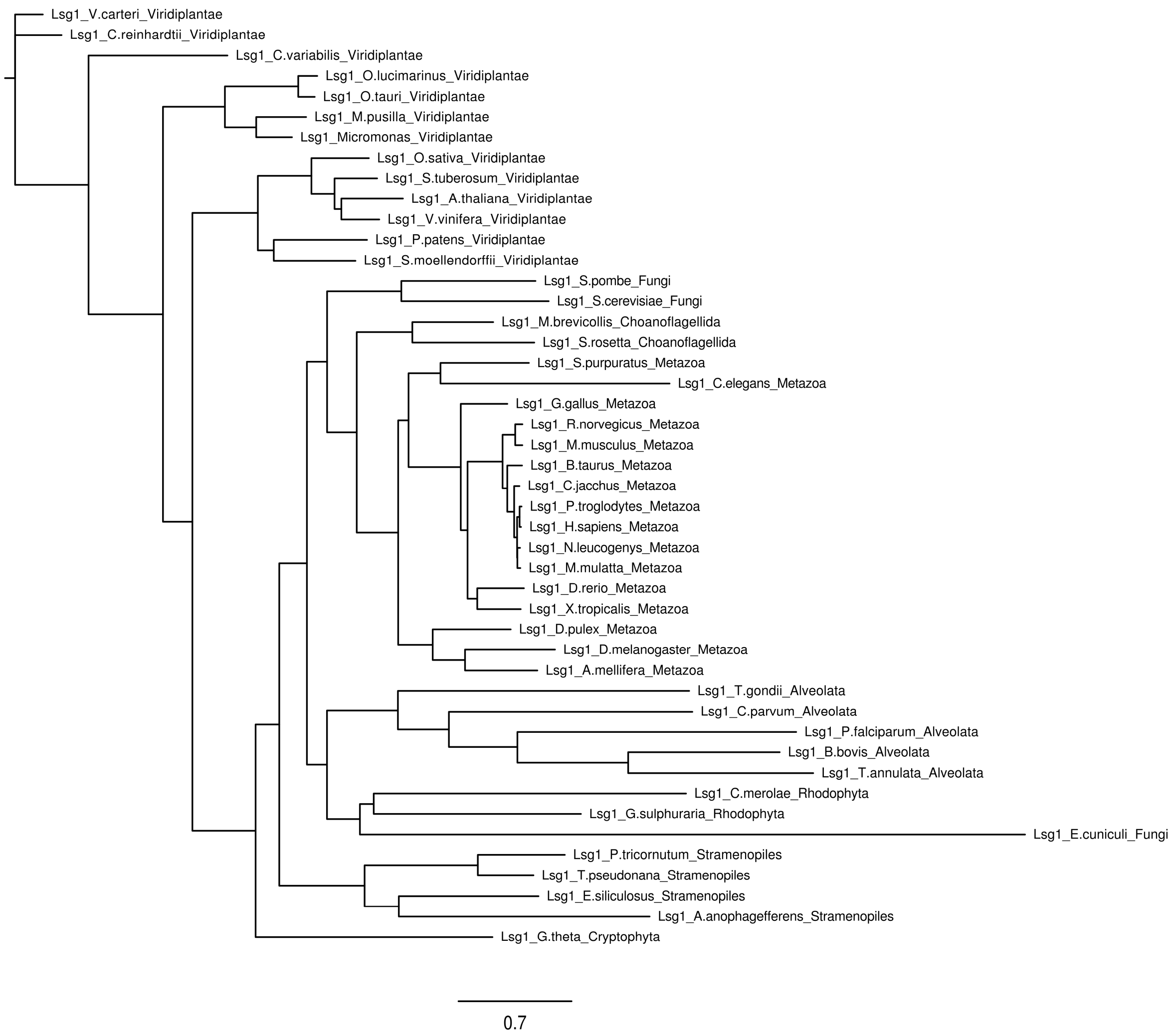

6) **Lsg1** subfamily tree

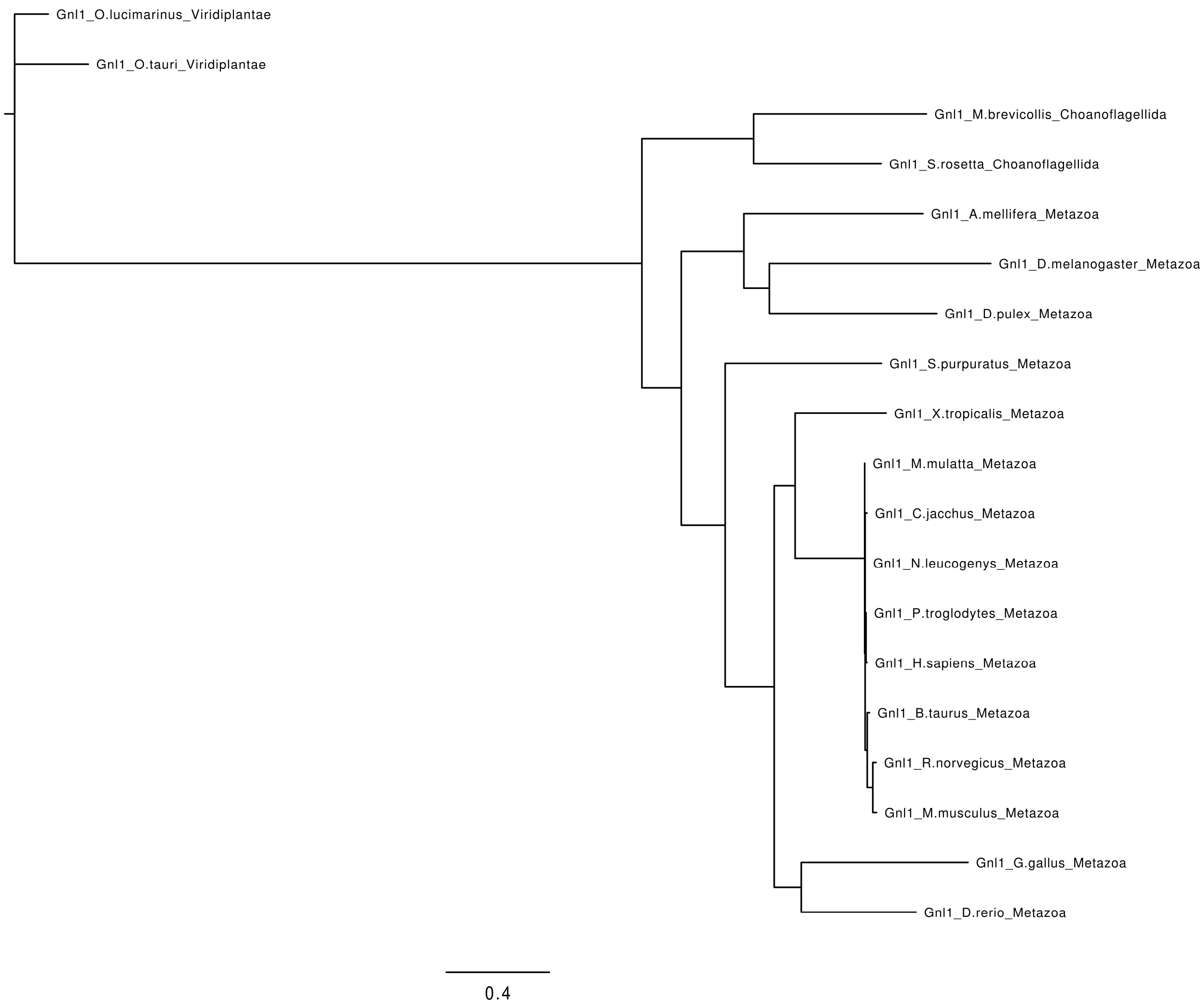

7) **Gnl1** subfamily tree

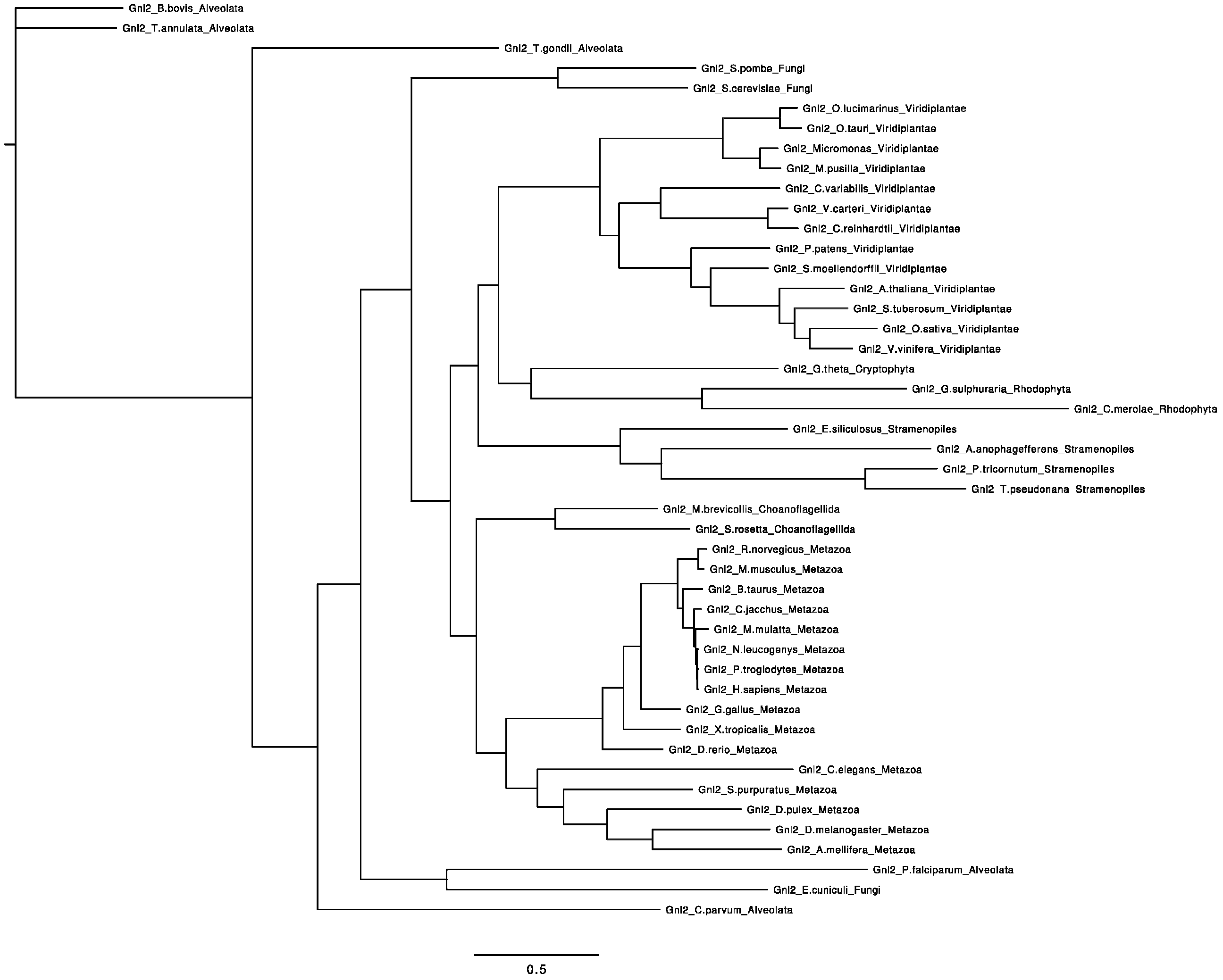

8) **Gnl2** subfamily tree

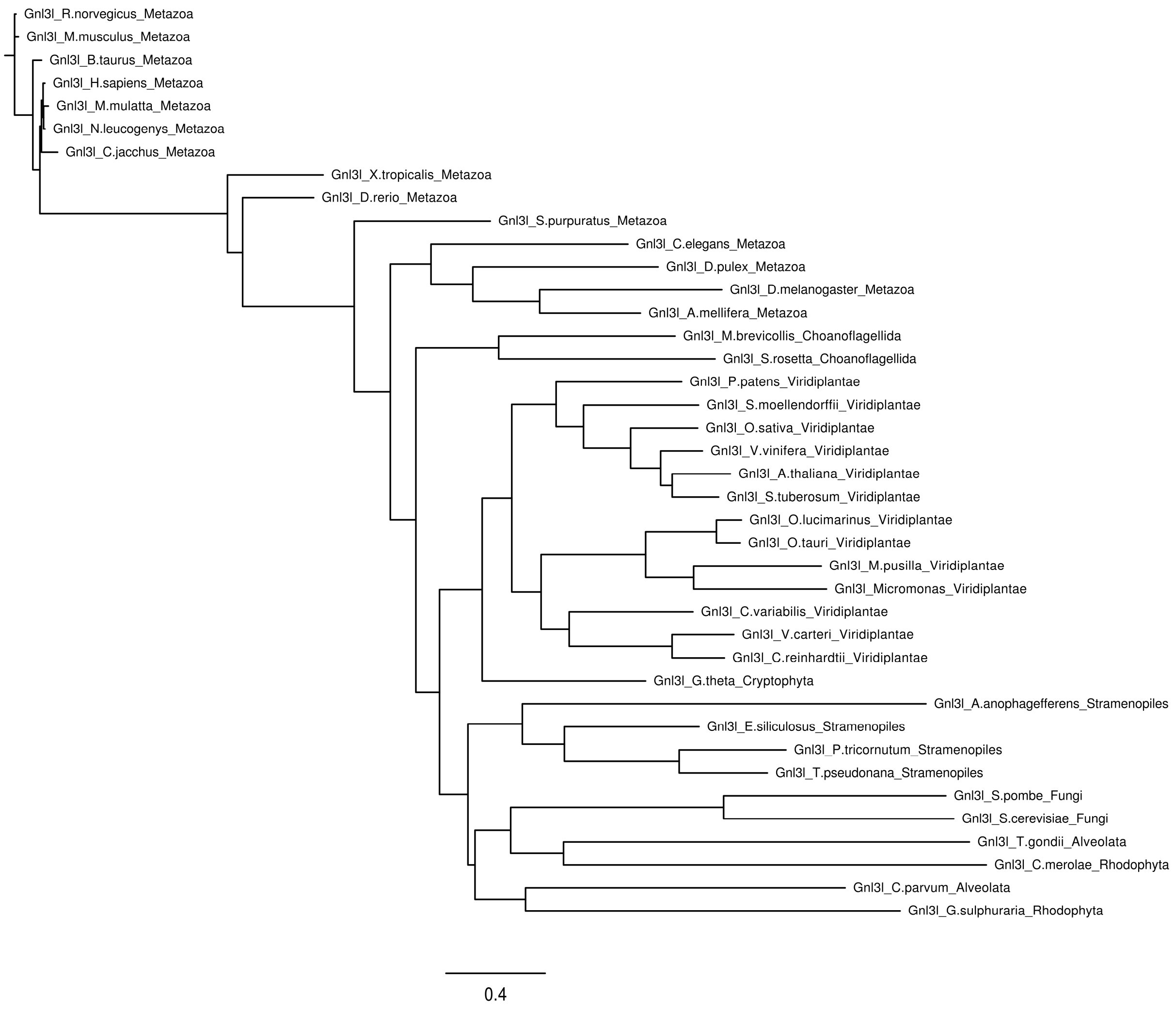

9) **Gnl3l** subfamily tree

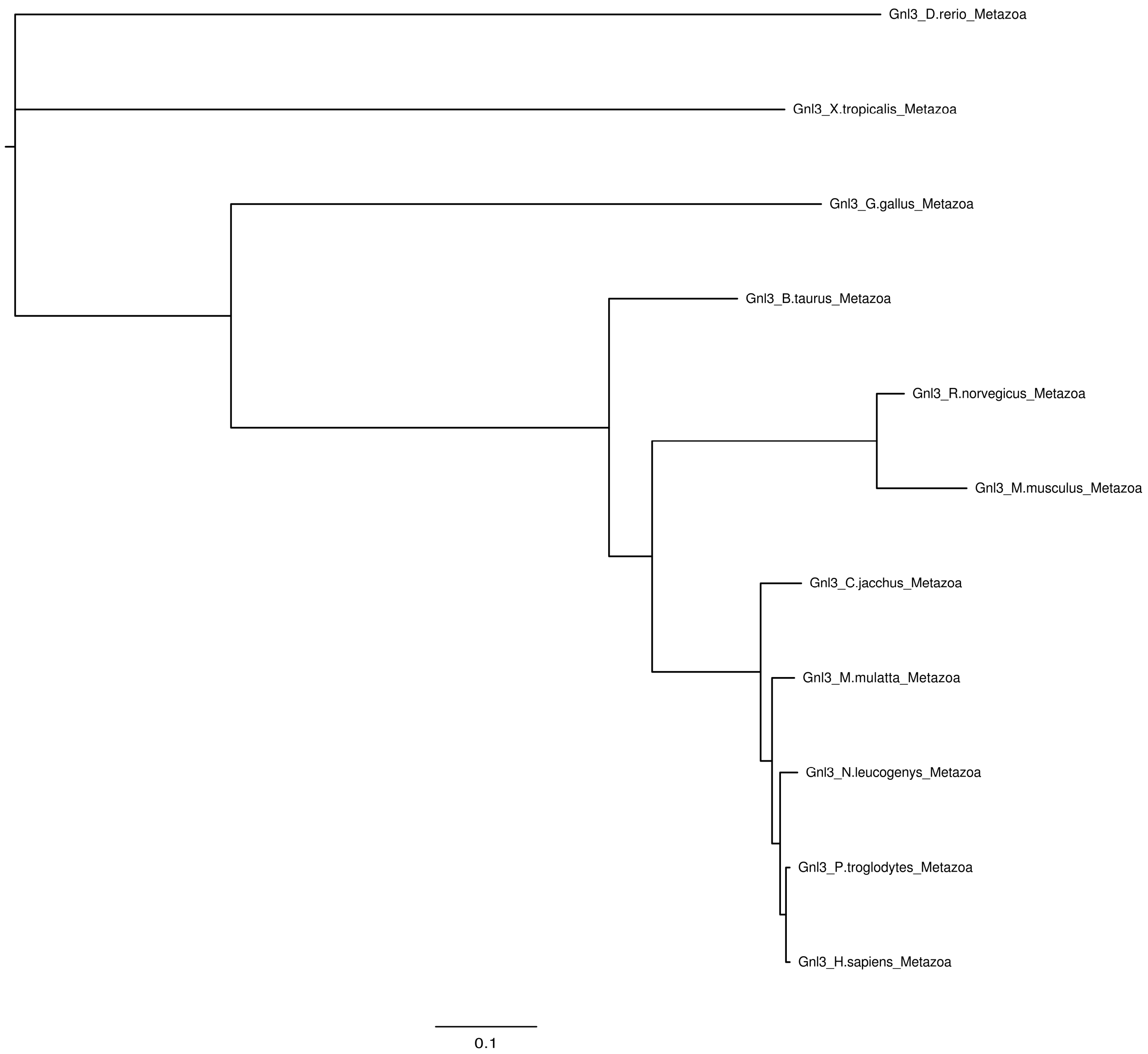

10) **Gnl3** subfamily tree
