# Supplementary material for: Reading the Evolution of Compartmentalization in the Ribosome Assembly Toolbox: The YRG Protein Family
Source: PLoS One. 2017 Jan 10;12(1):e0169750. doi: 10.1371/journal.pone.0169750 (PMC5224878; doi:10.1371/journal.pone.0169750)
Supplement: S5 File — The sequences are disposed in ten branches, one for each YRG protein subfamily: Gnl3, Gnl3l, Gnl2, Lsg1, Gnl1, YlqF, Mtg1, YjeQ, Noa1 and YAG. Main branches are labeled with a bootstrap support value (0–100), and the lowly supported ones are highlighted in red color. (PDF) [file pone.0169750.s005.pdf]

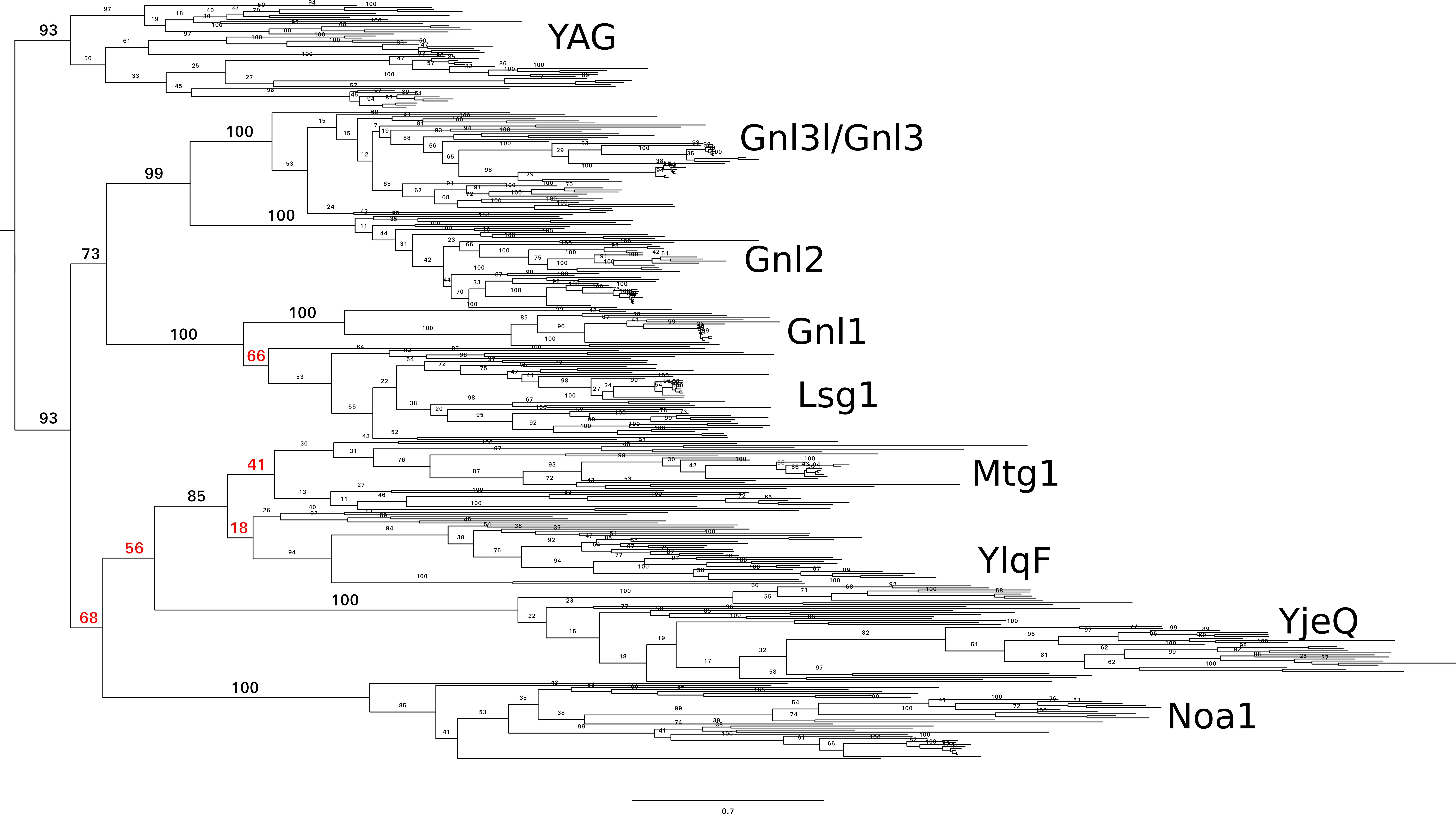
YAG
Gnl3l/Gnl3
Gnl2
Gnl1
Lsg1
Mtg1
YlqF
YjeQ
Noa1
0.7
